# Supplementary material for: Identification of Predictive Biomarkers of Lameness in Transition Dairy Cows
Source: Animals (Basel). 2024 Jul 10;14(14):2030. doi: 10.3390/ani14142030 (PMC11273747; doi:10.3390/ani14142030)

Figure S1. Comparison between cresol standard MS/MS spectrum (top chromatogram) with cresol MS/MS spectrum in the quality control (QC) sample (bottom chromatogram).

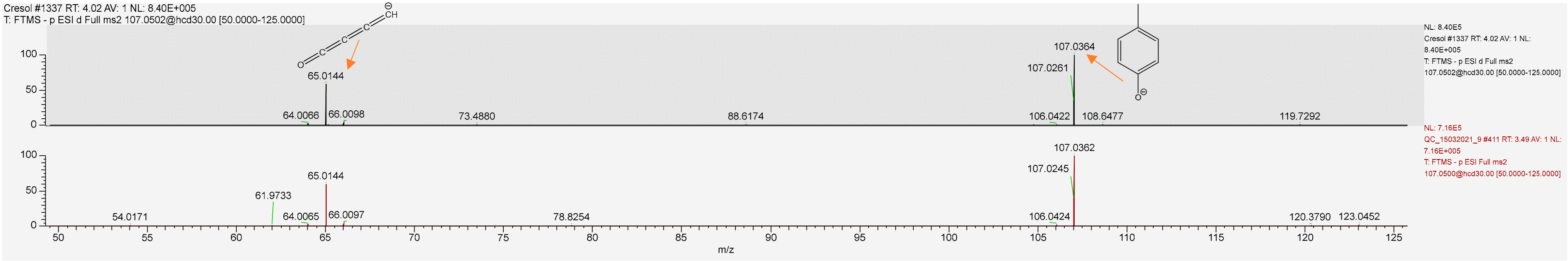

Figure S2. Comparison between gluconolactone standard MS/MS spectrum (top chromatogram) with gluconolactone MS/MS spectrum in the quality control (QC) sample (bottom chromatogram).

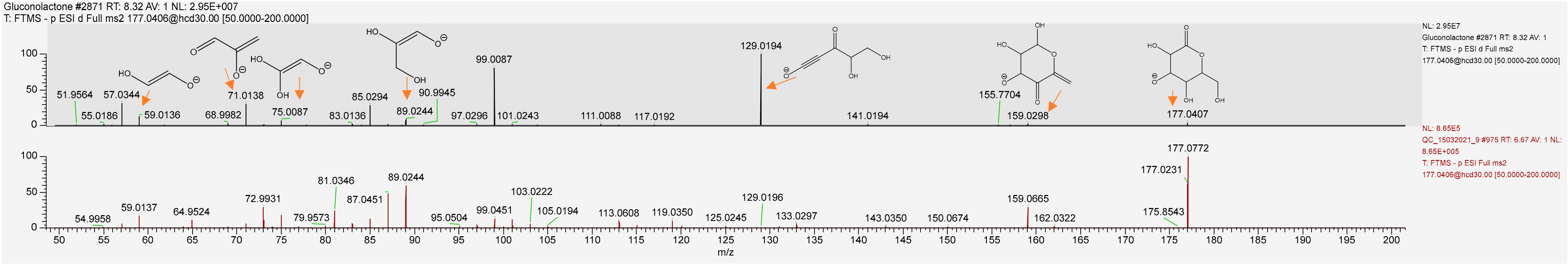

Supplement: Supplementary file 1 [file animals-14-02030-s001.zip › animals-3049091-supplementary.pdf]
